# First national analysis of severe obesity hospitalizations in Italy: insights from discharge card database

**DOI:** 10.3389/fpubh.2024.1332076

**Published:** 2024-02-23

**Authors:** Valerio Manno, Valentina Minardi, Maria Masocco, Francesco Cobellis, Giada Minelli, Donato Greco

**Affiliations:** ^1^Statistical Service, Istituto Superiore di Sanità, Rome, Italy; ^2^National Centre for Disease Prevention and Health Promotion, Rome, Italy; ^3^Department of Surgical, Oncological and Gastroenterological Sciences (DiSCOG), University of Padua, Padua, Italy; ^4^Cobellis Clinic Vallo Della Lucania (SA), Salerno, Italy

**Keywords:** hospital admission, burden, obesity, bariatric surgery procedure, abdominal reshaping surgery

## Abstract

**Background:**

Obesity is associated with numerous severe diseases necessitating intensive healthcare for diagnosis and treatment. Most patients with obesity, especially in its severe form, require at least one hospitalization. However, few studies in Italy have assessed the burden of obesity on the National Health System. This study aims to routinely estimate the ‘disease burden’ by analyzing hospital admissions related to severe obesity.

**Subjects:**

We analyzed the medical records of the Italian national hospital discharge database, including all patients older than 18 years discharged with the diagnosis of ‘severe obesity’.’ We included patients who underwent bariatric surgery, even without an explicit obesity code, such as laparoscopic restrictive gastric procedures, other stomach operations, and high gastric bypass. Special focus was given to those who underwent abdominal reshaping surgery. The cross-sectional survey PASSI in Italy served as an additional data source to estimate. The phenomenon was described using appropriate indicators, including rates and ratios between rates. Linear regression was employed to analyze trends in standardized rates over time.

**Results:**

Between 2014 and 2021, a total of 243,325 patients were discharged with a severe obesity code in Italy. Among these patients, 36.8% underwent at least one bariatric surgery procedure. We investigated the types of bariatric surgery procedures performed. The most frequent procedure was “other operations on the stomach,” where sleeve gastrectomy is included which also appears to be steadily increasing during the study period together with the gastric bypass, while the gastric bondage is decreasing over time.

**Conclusion:**

These findings underscore the significant burden of severe obesity on Italy’s healthcare system, a burden that is progressively increasing. The growing utilization of bariatric surgery suggests an escalating trend toward adopting drastic solutions to combat this health issue.

## 1 Introduction

Obesity is a pathological condition exerting major consequences on health and quality of life (1). Obesity is associated with an increased risk of serious noncommunicable diseases, such as diabetes, cardiovascular diseases, and certain cancer types (2–4). Moreover, during the coronavirus disease-2019 (COVID-19) pandemic, numerous studies revealed an association between obesity and severe COVID-19 (5, 6).

Obesity is partially preventable and decreasing its spread is one of the targets of the World Health Organization Global Action Plan for the Prevention of Noncommunicable Diseases (7). The current estimates of the Institute for Health Metrics and Evaluation reported excess weight, in general, to be responsible for 5.19 million deaths globally in 2019 (9% of all deaths) and over 40 million person-years lived with disabilities (Institute for Health Metrics and Evaluation). In Italy, it is estimated to be responsible for over 64 thousand deaths (or 10% of all deaths) and over 571,000 person-years lived with disabilities (8).

Excess weight and obesity in Italy’s adult population have been estimated for at least 20 years, with the most consolidated obtained from the periodic health survey conducted by the Italian Institute of Statistics (Istat) (9) and from the continuous surveillance system PASSI conducted by the Italian National Institute of Health (Istituto Superiore di Sanità, ISS) (10). Both are based on self-reported data during interviews administered to representative population samples, and several evaluation studies (11–15) have confirmed the validity of these methods, although they have emphasized a slight underestimation of the reported data compared to those directly measured on similar population samples.

Estimates from the ongoing Italian behavioral risk factor surveillance system PASSI show that 17 million adults were overweight in 2020–2021 and > 4 million were already obese (10).

Obesity is associated with several severe pathologies, thereby requiring intense healthcare for diagnosis and therapy, but most, if not all, patients with obesity will require at least one hospitalization in its severe form.

A positive correlation between obesity and healthcare utilization has frequently been reported (16), particularly with increasing physical disability or chronic diseases, and a meta-analysis also revealed an increase in healthcare services compared with non-obese population (17). Bariatric or weight loss surgery is an option for individuals with a body mass index (BMI) >40 or > 35 with comorbidities who are unable to lose weight by lifestyle modifications or pharmacotherapy (18, 19).

Italy has an information system on hospitalizations for >30 years, which collects information on any single discharge (ordinary or day-hospital regime) from public or private institutions through a standard national form (20).

In Italy, few studies attempt to measure the burden of obesity in the National Health System (21–23). The current study aimed to take ordinary estimates of the “disease burden” of these conditions by analyzing hospitalizations.

## 2 Materials and method

Obesity is defined as BMI (Kg/m^2^) where the thresholds adopted are: (1) underweight: BMI < 18.5; (2) normal weight: BMI = 18.5–25; (3) overweight: BMI = 25–30; (4) obese: BMI ≥ 30.0; and (5) severe obesity: BMI > 40.

### 2.1 Source of data

We analyzed records from the Italian National Hospital Discharge Database, which collects data from all patients discharged from an Italian hospital. Approximately 11 million forms annually of which approximately 8 million forms for ordinary discharge and approximately 3 million forms for day-hospital discharge. Anonymous demographic data (e.g., sex, date of birth, discharge date) as well as the primary diagnosis and up to five secondary discharge diagnoses are recorded, and diagnoses are codified at the hospital level according to the World Health Organization (WHO) International Classification of Diseases, Ninth Revision, Clinical Modification (ICD-9-CM) (24).

The study population is patients aged ≥18 years dismissed from January 2014 to December 2021.

### 2.2 Hospitalization for severe obesity

This study included all patients >18 years of age discharged with ICD-9-CM diagnosis code 278.01 (severe obesity) as a primary or secondary diagnosis. Further. we included patients undergoing bariatric surgery even without an obesity code (i.e., with code 44.95: laparoscopic gastric restrictive procedure, 44.99: other operations on stomach, and 44.31: high gastric bypass) as primary or secondary procedures. Within those patients, a focus was made on those who underwent abdominal moderation surgery (code: 86.83) as primary or secondary procedures.

We identified patients with first admission during 2014–2021 and used a “wash-out” period from 2001 to 2013 to identify and exclude cases with previous diagnoses of severe obesity or undergoing bariatric surgery.

In addition to the analyses conducted on individual first admissions of patients, all hospitalizations that occurred between 2014 and 2021 were considered as the totality of the phenomenon. This enabled the calculation of average days of hospitalization as the difference between the date of discharge and the date of admission for ordinary admissions.

### 2.3 The self-reported national surveillance system (PASSI)

The PASSI was used as an additional source to estimate the burden of severe obesity in Italy. PASSI is an ongoing cross-sectional survey in Italy that monitors the prevalence of the main behavioral risk factors for chronic non-communicable diseases. It is coordinated by the ISS (25) and conducted by regional and local health units. The survey aimed to describe the health profile of the general population, plan prevention, and health promotion interventions, and monitor their effectiveness over time. The PASSI sample is randomly selected from a list of residents and categorized by gender and age. Specially trained personnel used a standardized questionnaire to conduct telephone interviews. The data collected are anonymized, recorded in a national database, and aggregated into annual and four-year data sets to guarantee an adequate sample size for subgroup analyses. The PASSI methods for data collection have been described in more detail elsewhere (26, 27).

Self-reported weight and height by interviewed were used to calculate BMI; people with BMI over than 40 was cathegorized as severe obese.

### 2.4 Statistical methods

We used the direct standardization method, taking the 2013 European standard population as a reference, to calculate age-standardized first hospitalization rates. The age and gender distribution were described, the ratios of the crude rates within age groups and sexes (RR) and between the numerosity of the two genders (sex ratio) were calculated, and the Kruskal-Wallis test was used to assess gender and age differences and geographical distribution. Linear regression was used to analyze trends in standardized rates over time, the parameter estimates and R-square of which are reported. SAS version 9.4 software was used for statistical analysis.

## 3 Results

**Figure 1:**
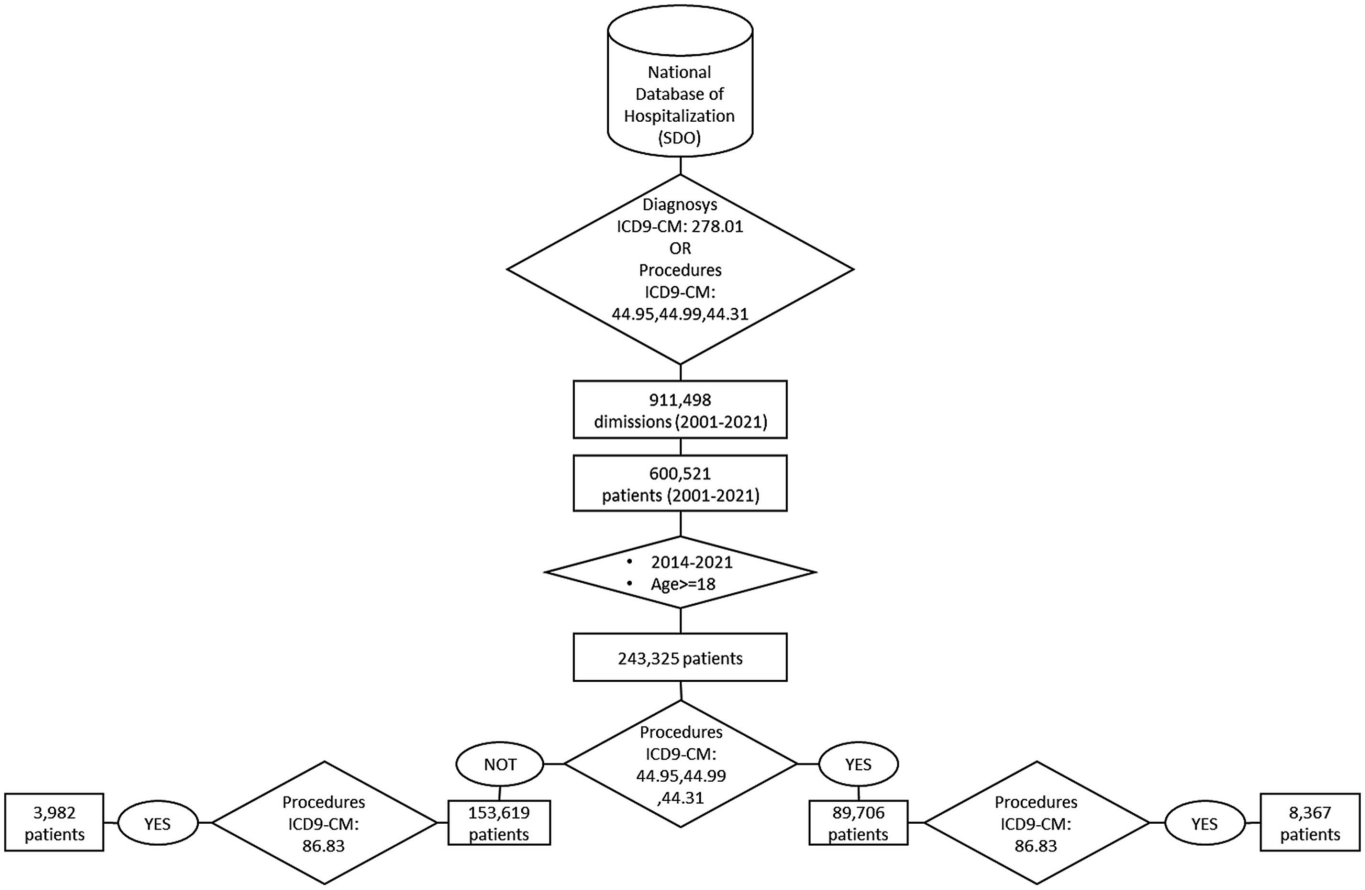
Logical order for selecting severe obesity cases and patients who underwent bariatric surgery.

Figure 1 shows the data extraction procedure.

This study analyzed the period from the coronavirus disease 2019 (COVID-19). Notably, in 2020, due to the first COVID-19 pandemic wave, the number of hospital discharges decreased by approximately 20% compared to 2019. In 2021, the number of hospital discharges increased, which was almost similar to that of pre-pandemic years. Additionally, it could have been because hospitalizations that were planned in 2020 were postponed to 2021.

### 3.1 Patients diagnosed with severe obesity

A total of 243,325 patients aged ≥18 years were discharged with the code severe obesity from 2014 to 2021. Of them, 67% were women, with a sex ratio of W/M = 2.02 (95% confidence interval [CI]: 2.01–2.03). The age distribution groups are similar by gender (*p* = 0.2482) with a woman-disadvantaged risk in the younger and older groups. The geographical distribution of cases does not vary by gender (*p* = 0.0761). The regions with the highest standardized rates of severe obesity-related first hospitalization are located in the south of Italy, including Campania, Apulia, Basilicata, and Sicily (Table 1).

**Table 1:** Distribution by gender of severe obesity cases: age groups, region of residence: observed and standardized hospitalization rates (per 100 thousand inhabitants) Italy, 2014–2021.

	Men		Women	
	Observed	SR	Observed	SR
Age groups				
18–49	34,558	36.08	81,370	86.34
50–64	25,293	50.54	42,059	79,82
65–79	16,797	48.40	27,508	68.46
80+	3,949	31.98	11,791	55. 38
Regions				
Piemonte	5,362	31.07	12,232	69.85
Valle d’Aosta	233	45.86	390	75.29
Lombardia	8,537	22.03	21,283	54.54
P.A. Bolzano	386	19.31	694	33.27
P.A. Trento	482	22.71	885	40.39
Veneto	4,318	22.25	8,422	42.73
Friuli	1754	34.87	2,851	55.45
Liguria	1,657	26.53	3,843	60.9
Emilia-Romagna	5,702	32.36	11,061	59.91
Toscana	5,009	33.42	10,026	62.18
Marche	1,078	30.98	2,107	56.27
Umbria	1,623	26.38	2,757	41.92
Lazio	8,223	37.36	18,591	79.81
Abruzzo	1,570	30.22	3,133	57.71
Molise	459	36.33	715	53.08
Campania	13,286	61.27	24,247	106.26
Puglia	7,599	49.26	14,241	86.53
Basilicata	1,195	52.9	1775	72.41
Calabria	2,342	31.49	3,937	49.63
Sicilia	7,471	39.84	14,758	74.64
Sardegna	2,311	34.61	4,780	68.43

**Figure 2:**
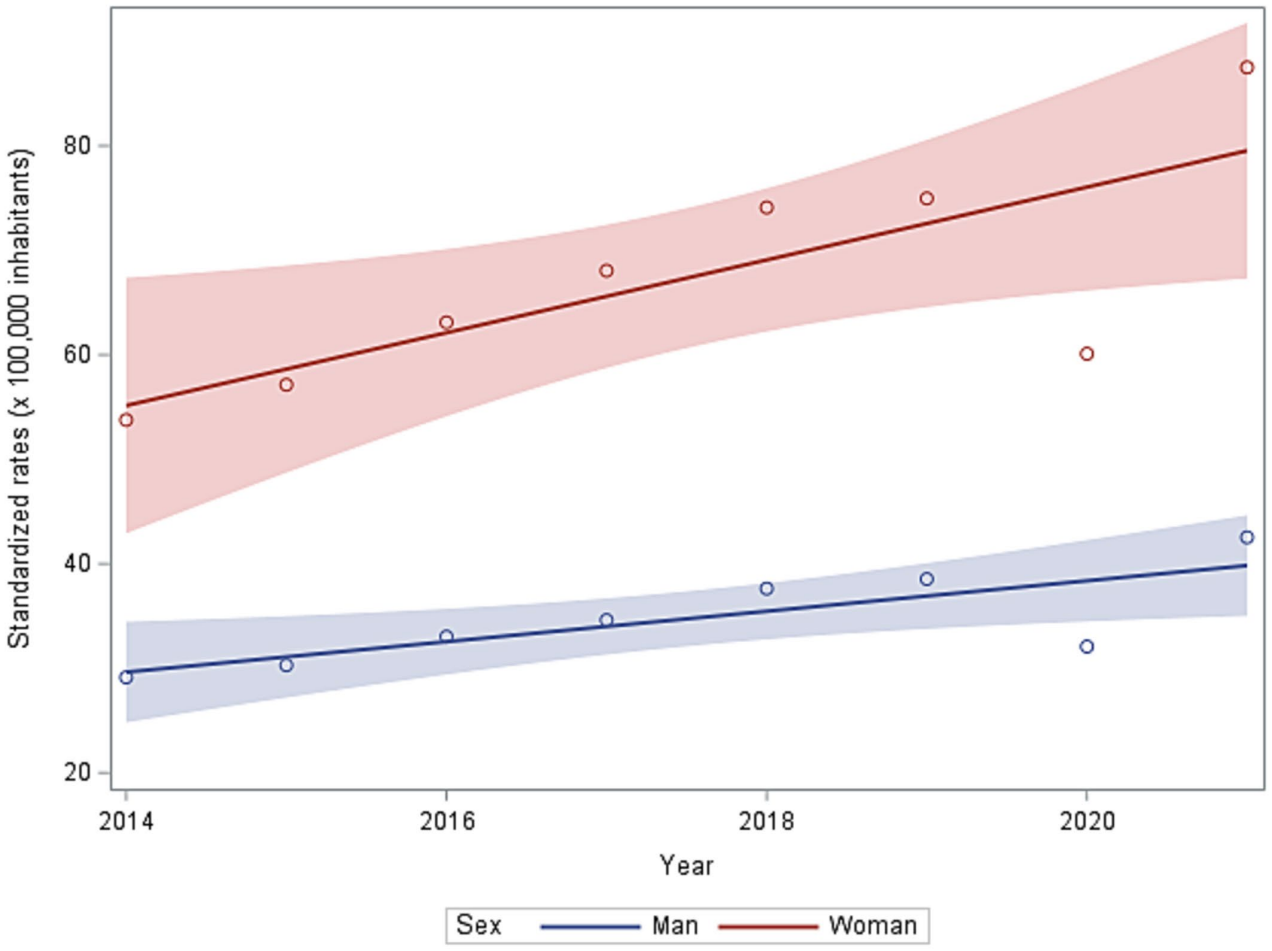
Standardized rates (per 100 thousand inhabitants) of first hospitalization in 2014–2021 in men and women.

Severe obesity in first hospitalized patients showed an overall increasing trend from 2014 to 2021 (Figure 2), with higher rate values in s with a stronger trend (Parameter estimated: man: 1.46 Man [R-square: 0.62]; woman: 3.84 [R-square: 0.59]).

From 2014 to 2021, 84, 12, and 4% of patients had one, two, and three hospitalizations, respectively.

### 3.2 Patients with severe obesity and bariatric surgery procedure codes

Of the patients with severe obesity, 36.8% (89,706 out of 243,325 patients) underwent at least one bariatric surgery procedure. Bariatric surgery was more frequent among women, with a sex ratio of W/M = 1.65 (95% IC: 1.64–1.66) and W/M = 2.97 (95% IC: 2.94–3.01) for those with obesity who have none and at least one bariatric surgery procedure, respectively.

The age distribution is similar for men and women, both for those with severe obesity who have none (*p* = 0.1489) and at least one bariatric surgery procedure (*p* = 0.3865), respectively. Patients aged 18–49 years with severe obesity who have at least one bariatric surgery procedure accounted for >70%.

**Figure 3:**
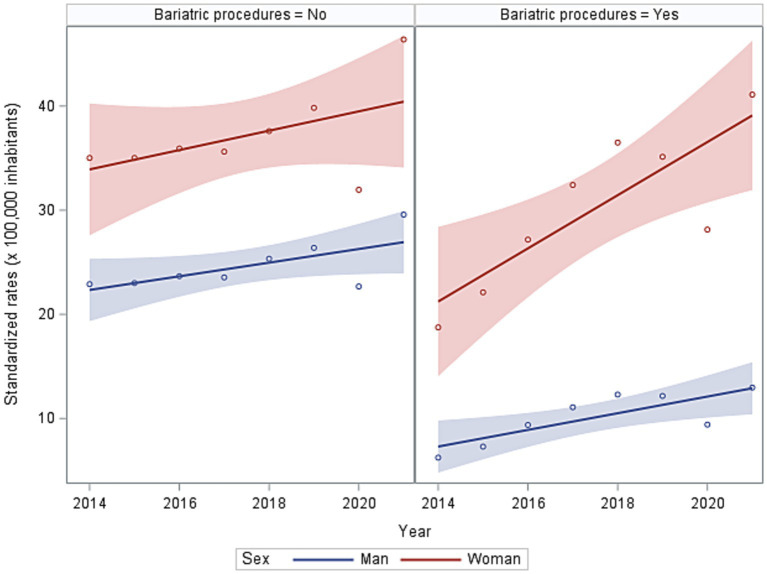
Trends in standardised rates of first hospitalisation for severe obesity with or without bariatric procedure in 2014–2021.

Of the patients with severe obesity, 28 and 41% of men and women, respectively, underwent at least one bariatric surgery, but with no significant difference for the first two age groups. An overall increasing trend of the first severe obesity hospitalization rate was observed from 2014 to 2021 (Figure 3). This phenomenon is reflected in the increase of cases with bariatric surgery, and the growth is more pronounced in women.

**Figure 4:**
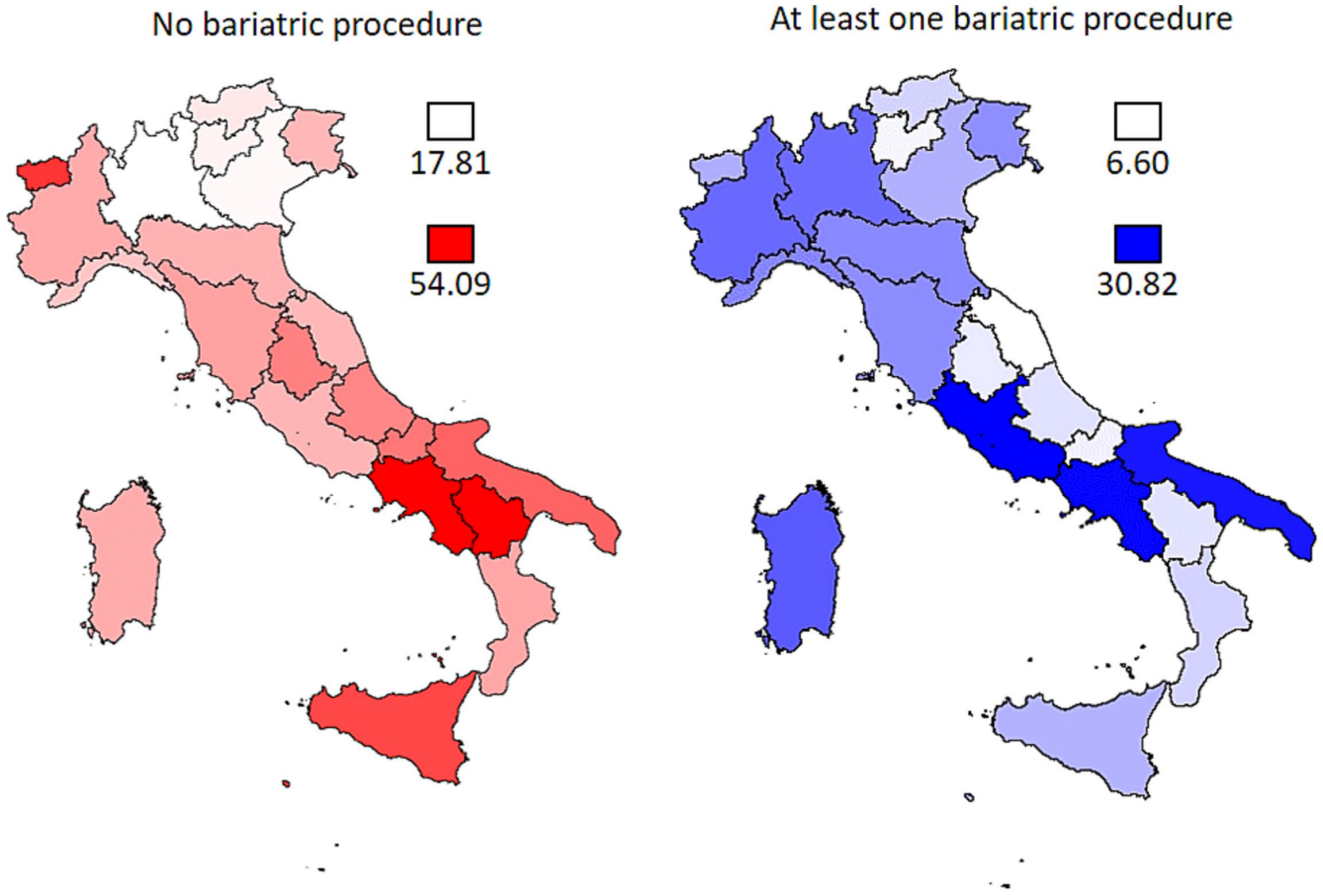
Regional standardised rates (×100,000 population) of first admission for severe obesity with or without bariatric procedure in 2014–2021.

Figure 4 shows that the severe obesity-related first hospitalization rates by region of residence still show higher rates in the south of Italy as in Figure 3, but more distributed rates for those that underwent surgery.

### 3.3 Patients who underwent abdominal reshaping surgery

A total of 12,349 patients underwent abdominal reshaping surgery, i.e., 5% of first hospitalizations for severe obesity and 2.5% of first hospitalizations for bariatric surgery. The rate ratio between the proportions of woman versus man first hospitalizations indicates that women have a 3.37% higher risk of undergoing remodeling surgery than men.

Remodeling required one, two, and more hospitalizations in 82, 13, and 4% of men and 70, 20, and 9% of women, respectively.

### 3.4 All hospitalizations for severe obesity with or without bariatric surgery

Considering all the hospital discharges and not individual patients with or without bariatric surgery, from 2014 to 2021, 378,491 discharges of patients aged −18 years were documented, including 355,637 in ordinary regimes and 22,854 in day-hospital regimes.

Approximately 32% (112,522) of all ordinary discharges are referred to discharges in ordinary regimes for bariatric surgery (Table 2). An overall increasing trend of hospital discharge for bariatric surgery was documented from 2014 to 2021 (more than doubling in 8 years), and this phenomenon is more pronounced in s. The average hospital stay decreased steadily over the study period for both genders.

**Table 2:** Number of hospitalizations for the ordinary regime and average length of stay (LS) for gender and year of hospitalization with or without bariatric surgery Italy, 2014–2021.

Year	With bariatric surgery				Without bariatric surgery			
	Man		Woman		Man		Woman	
	*N*°	LS	*N*°	LS	*N*°	LS	*N*°	LS
2014	1951	6.18	6,307	4.85	9,869	7.90	18,116	7.85
2015	2,304	6.14	7,559	4.76	10,111	7.71	18,915	7.68
2016	3,112	5.41	9,367	4.38	10,691	7.75	19,556	7.61
2017	3,710	5.03	11,162	4.24	10,800	7.83	19,983	7.68
2018	4,267	4.63	12,703	4.1	11,437	7.86	20,713	7.60
2019	4,669	4.47	13,433	3.78	11,995	7.79	21,380	7.55
2020	3,279	4.29	9,820	3.71	9,746	8.90	16,453	8.02
2021	4,544	4.07	14,335	3.41	12,007	9.26	21,343	8.03

Considering the number of discharges in ordinary regimes and not individual patients without bariatric surgery, from 2014 to 2021, 265,832 hospital discharges were performed. The number of discharges does not change during the study period for both genders. The average length of hospitalization stays in ordinary regimes does not vary from 2014 to 2019, while there is an increase from 2020, especially for men. Hospitalization for patients undergoing bariatric surgery is less than half of those without surgery. The differences between the average length of hospitalization with and without bariatric surgery are statistically significant (*p* < 0.001).

### 3.5 Type of surgery of patients undergoing bariatric surgery

The type of bariatric surgery procedures was investigated (Table 3). The most frequent bariatric surgery procedure is the one corresponding to ICD9-CM code 44.99, or “other operations on the stomach,” where sleeve gastrectomy is included which also appears to be steadily increasing during the study period together with the gastric bypass, while the gastric bondage is decreasing in time. In 2021, 73% of interventions were sleeve gastrectomy, 14.3% were gastric bypass, and 12.7% were gastric bondage. Different proportions of those from the previous 8 years (8.6% sleeve, 38.2% bondage, and 13.1% bypass) show a significant change in time. The gastric bondage has a significantly shorter duration than the other two operations.

**Table 3:** Number of hospitalizations for the ordinary regime and average length of stay (LS) for bariatric procedure by year and gender Italy, 2014–2021.

Year	Laparoscopic gastric surgery				Other operations on the stomach				High gastric bypass			
	(gastric bondage)				(sleeve gastrectomy)				(ICD9-CM: 44.31)			
	(ICD9-CM: 44.95)				(ICD9-CM: 44.99)							
	Man	LS	Woman	LS	Man	LS	Woman	LS	Man	LS	Woman	LS
2014	600	3.35	2,586	3.11	1,064	7.34	2,988	5.86	312	7.67	784	6.91
2015	604	3.47	2,525	3.3	1,405	6.85	4,126	5.22	313	8.11	979	6.66
2016	658	3.78	2,620	3.3	2,121	5.54	5,628	4.61	364	7.65	1,205	5.77
2017	577	4.11	2,431	3.31	2,700	4.94	7,387	4.26	468	6.81	1,507	5.77
2018	560	3.76	2,476	3.62	3,191	4.6	8,683	4	579	5.77	1807	5.43
2019	474	3.95	2,319	3.29	3,586	4.33	9,407	3.68	698	5.71	2049	4.92
2020	349	3.27	1,664	3	2,487	4.32	6,878	3.73	510	4.91	1,547	4.61
2021	420	3.29	2035	3.11	3,524	4.12	10,507	3.37	674	4.31	2058	4.09
Total	4,242	3.65	18,656	3.27	20,078	4.88	55,604	4.08	3,918	6.03	11,936	5.28

### 3.6 Burden of hospitalizations among people severely obesity

Estimates from the self-reported national surveillance system PASSI for 2016–2021 (Table 4) report a 0.56% prevalence of severe obesity in Italy (annual average: 95% IC: 0.53–0.59%; from 0.52% in 2016 to 0.59% in 2021). An estimated 294,322 individuals had severe obesity in 2021 (the Italian population over the age of 18 years is 49,885,100 in 2021).

**Table 4:** Prevalence of severe obesity in individuals in the surveillance system PASSI (ISS) in 2016–2021.

Age group, year	Prevalence	95% Confidence interval	
18–49	0.38%	0.34%	0.42%
50–64	0.77%	0.70%	0.85%
65–79	0.71%	0.65%	0.78%
80+	0.43%	0.35%	0.52%
Total	0.56%	0.53%	0.59%

During our study period, 243,325 severe obesity-related first hospitalizations were documented (30,416/year), approximately 10.3% of the total severe obesity population. This number is growing, as is the use of surgery for treatment (Figure 4).

## 4 Discussion

This study revealed the first original analysis of hospitalization data for severe obesity at the national level in Italy, which is the discharge card database that collects every hospitalization continuously.

To date, most studies that have produced estimates of severe obesity have been based on population surveys with interview data (11–14, 23), while area- and time-limited case studies on individual patients have been lacking.

The discharge records refer to patients aged ≥18 years who were hospitalized and diagnosed based on objective clinical measures. The study identified new individuals who were hospitalized for severe obesity. This study found >243,000 people who were discharged with a diagnosis of severe obesity, with >30,000 annually but with a marked upward trend over the 8 years of the study period.

The same patients benefited from >379,000 hospitalizations with an average bed occupancy per admission of >6 days with an estimated consumption of >2.5 million hospital days.

Estimates of severe self-reported obesity in the PASSI system for 2021 give a prevalence estimate of 279,294,000 people. Approximately >1 in 10 people in the same year were hospitalized. Therefore, most people with severe obesity do not resort to interventions to mitigate their health problems although obesity is a complex health disorder that significantly increases the risk of several chronic diseases. A recent Italian study estimated that the total costs associated with obesity in Italy amounted to €13.34 billion in 2020. Direct costs were €7.89 billion, with cardiovascular diseases having the highest impact on costs (€6.66 billion), followed by diabetes (€0.65 billion), cancer (€0.33 billion), and bariatric surgery (€0.24 billion) (21).

The trend of hospitalization is steadily increasing, more marked among women, who are twice as likely as men to be hospitalized for severe obesity, and with more young adults (18–49 years). Southern Italy has substantially higher rates of hospitalization with a record in the Campania Region in agreement with self-reported estimates.

The problem of obesity in the region is of greater concern as it affects not only the adult population but also children and adolescents. The Campania region holds the highest number of children with overweight and obesity in Italy, with 44% overweight (28). Therefore, specific actions and coordinated management should be initiated in this region as soon as possible to ensure the mitigation and decrease of this phenomenon. Increasing awareness of the problem through school education is crucial among these actions.

One-third of patients undergo bariatric surgery, with a clear increase in the time trend and woman contribution. Patients who underwent an operation demonstrated a length of hospitalization of up to half the average length of stay of non-operated patients in recent years.

Sleeve gastrectomy is the laparoscopic operation of choice, accounting for approximately 75% of all operations in 2021, with 2.5% of hospitalized patients undergoing abdominal reshaping, and 2 out of 10 undergoing more than one operation. Laparoscopy has revolutionized abdominal surgery over the past 30 years. Globally, 15 million laparoscopic surgical operations are performed annually (29).

Thinking that severe obesity is not significantly amenable to dietary or pharmacological prevention strategies, while it becomes amenable to surgery, seems logical. Bariatric surgery is the most clinically effective treatment for persons with severe and complex obesity but with less clear psychosocial outcomes. Follow-up care after bariatric surgery is known to be important, but only a few studies reported on the follow-up of patients who have undergone this surgery (15). According to recent evidence, Obesity often together with hyperglicemia and metabolis syndrome is a negative prognostic factor in breast cancer and also cardiac dysfunction then increasing hospitalization in these patients (30).

This indicates that prevention and treating obesity in vulnerable patients like cancer nes, possibly attending to WCRF guidelines (31) could significantly reduce hospitalizations and severe outcomes of these patients.

This study has significant limitations. Hospital discharge forms do not allow us to know the real “medical history” of patients with obesity, but we are confident that patients belonging to both subgroups described in our work (those with hospitalization diagnosed with severe obesity and those with bariatric surgery, with or without severe obesity diagnosis) can be defined as severely obese by investigating the diagnosis and procedure codes.

Considerably, in 2020, in the acute phase of the COVID-19 pandemic, the total number of hospitalizations demonstrated a 20% decrease, compared to 2019. In 2021, the total number of hospitalizations increased, getting closer to the previous years; however, it should be attributed to a delay in hospitalizations, which causes biased results.

We have verified that the national database on hospitalizations enables the identification of “incident” cases as first recurrence by cause, and would therefore also allow, with appropriate further studies, the construction of a history of hospitalizations per individual patient allowing outcome estimates of bariatric interventions over time.

## 5 Conclusion

These data highlight a significant burden of severe obesity on the country’s healthcare system with an upward trend indifferent to the many national and local initiatives to prevent and combat obesity. The parallel growth in the use of bariatric surgery indicates an increasing use of a drastic solution to the problem.

## Data availability statement

The datasets presented in this article are not readily available because the data analysis used in this study complies with the European General Data Protection Regulation (EU GDPR2016/679). The dataset of hospital discharge forms is owned by the Italian Ministry of Health. The Italian Data Protection Authority authorized the processing of personal data by the Istituto Superiore di Sanità for reasons of public interest in the field of public health. The analyses presented in the article are based on aggregated data. Requests for access to the hospital discharge dataset should be addressed to: GM, giada.minelli@iss.it.

## Ethics statement

The studies involving humans were approved by National ethics committee for clinical trials of public research bodies (EPR) and other national public institution (CEN). The studies were conducted in accordance with the local legislation and institutional requirements. Written informed consent for participation was not required from the participants or the participants’ legal guardians/next of kin in accordance with the national legislation and institutional requirements.

## Author contributions

VMa: Conceptualization, Data curation, Formal analysis, Investigation, Methodology, Software, Writing – original draft. VMi: Data curation, Formal analysis, Investigation, Methodology, Software, Writing – original draft. MM: Data curation, Formal analysis, Investigation, Methodology, Writing – original draft, Writing – review & editing. FC: Conceptualization, Supervision, Writing – review & editing. GM: Conceptualization, Investigation, Project administration, Supervision, Validation, Writing – original draft, Writing – review & editing, Funding acquisition, Resources, Visualization. DG: Conceptualization, Supervision, Validation, Writing – original draft, Writing – review & editing.
